# Digesting the alphabet soup of LCA

**DOI:** 10.1007/s11367-018-1478-0

**Published:** 2018-05-23

**Authors:** Jeroen B. Guinée, Stefano Cucurachi, Patrik J.G. Henriksson, Reinout Heijungs

**Affiliations:** 1 Institute of Environmental Sciences (CML), Department of Industrial Ecology, Leiden University, Einsteinweg 2, 2333 CC Leiden, The Netherlands; 2 Stockholm Resilience Centre, Stockholm University, 10691 Stockholm, Sweden; 3 Department of Econometrics and Operations Research, Vrije Universiteit Amsterdam, De Boelelaan 1105, 1081 HV Amsterdam, The Netherlands

**Keywords:** Attributional LCA, Consequential LCA, Explorative LCA, Modes of LCA, Modelling

## Introduction

One of the most pertinent and enduring debates within the life-cycle assessment (LCA) community is on consequential (CLCA) and attributional LCA (ALCA), see for example, Weidema et al. (1999), Guinée et al.(2002), Ekvall and Andræ (2006), Schmidt (2010), Zamagni et al. (2012), Rehl et al. (2012), Anex and Lifset (2014), Brandão et al. (2014), Suh and Yang (2014), Dale and Kim (2014), Hertwich (2014), Plevin et al. (2014a), Plevin et al. (2014b), Ekvall et al. (2016), and Weidema et al. (2018). Amongst other things, the debate focuses on similarities and differences between these two modes and on which mode is more appropriate for which case or question. Some authors claimed superiority of one of these modes over the other (Plevin et al. 2014a; Weidema et al. 2018). Here, we refrain from further dwelling on this discussion, but rather discuss the more recent emergence of other modes of LCA. These new modes have been developed by independent scholars, outside the ISO standards (International Organization for Standardisation 2006) and outside national and continental guidelines (e.g., European Commission—Joint Research Centre—Institute for Environment and Sustainability 2010). They all focus on estimating life-cycle impacts of future systems and we argue that they are all varieties of analysis fitting under the umbrella of “explorative” LCA. To substantiate this, we first look into the definitions of ALCA, CLCA, and some of the newer modes of LCA. We then briefly discuss similarities and differences between these different LCA modes, and conclude that rather than discussing different modes, the LCA community should focus on discussing their underlying real differences.

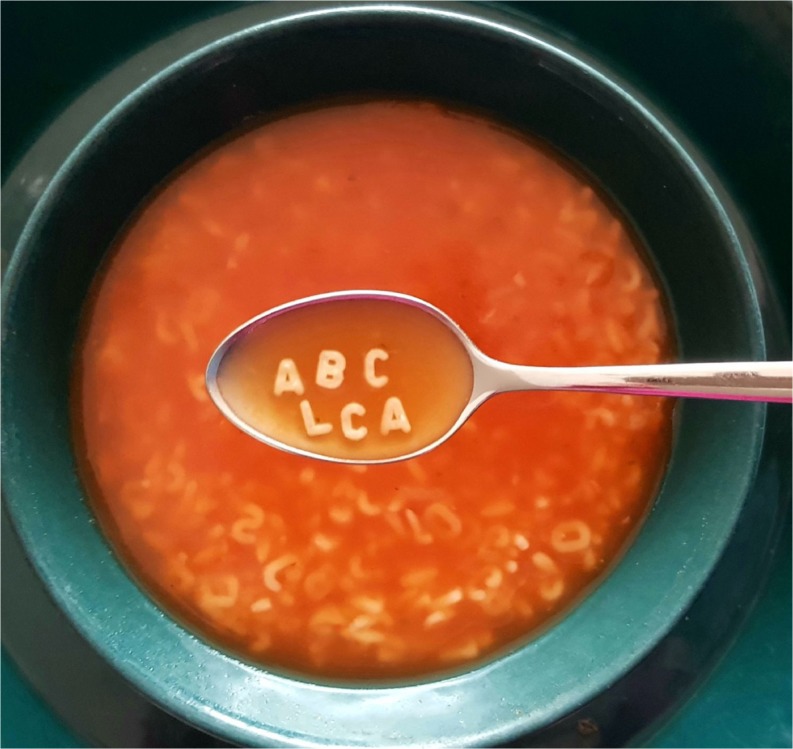



## Panoply of definitions

A widely used set of definitions for ALCA and CLCA was provided in the UNEP report on “Global Guidance Principles for Life Cycle Assessment Databases” (UNEP 2011):

- Attributional LCA (ALCA): to provide information on what portion of global burdens can be associated with a specific product life cycle.
- Consequential LCA (CLCA): to provide information on the environmental burdens that occur, directly or indirectly, as a consequence of a decision (usually represented by changes in demand for a product).

In the recent LCA literature, we can find many more modes of LCA. Below, we list these other modes of LCA and provide a brief definition:

- Backcasting LCA (BLCA): exploring ways—in a life-cycle perspective—to meet normatively defined sustainability levels (planetary boundaries) through adapted affluence (as consumption levels), population growth, and/or technologies (Heijungs et al. 2014);
- Decision LCA (DLCA): based on CLCA but using the actual or anticipated financial and contractual relations between economic actors (business-to-business relations) as the main basis of information (Frischknecht 1998; Frischknecht and Stucki 2010);
- Integrated LCA (ILCA): LCA integrated with other modeling approaches such as input-output analysis, energy-scenario modeling, and, for example, material flow analysis (Hertwich et al. 2014); method for assessing the environmental and resource implications of scenarios for large-scale adoption of climate change mitigation measures (Gibon et al. 2015);
- Anticipatory LCA (NLCA): a forward-looking, non-predictive tool that increases model uncertainty through inclusion of prospective modeling tools, decision theory, and multiple social perspectives (Wender et al. 2014);
- Prospective LCA (PLCA): estimating future life-cycle environmental impacts using scenarios (Spielmann et al. 2005; Walser et al. 2011);
- Scenario-based LCA (SLCA): LCA based on scenarios separating three modeling processes, life-cycle modeling, scenario modeling, and valuation modeling (Fukushima and Hirao 2002).

Perhaps, there are a few more varieties that we missed. But the conclusion is clear: an alphabet soup of LCA modes has emerged. The big question is of course: what is their relation?

## Similarities and differences

ALCA is the only mode focusing on modeling a situation as it is, either in the past, present, or future, but without any changes. The other modes, from BLCA up to SLCA, have a lot in common. They all aim at estimating the effects of changed situations, where the change and/or the background state are based on a scenario.

There are of course also differences between these modes regarding questions addressed (see Table 1 for some selected examples from literature), objects of analysis, processes involved, object of analysis, temporal scope, processes covered, data scope, additional methods and models adopted, and allocation methods used; see Table 2 for a summary of similarities and differences.

**Table 1:** Examples of questions addressed by different modes of LCA

	Question	Reference
ALCA	What is the life-cycle impact of 1 kWh of electricity at grid in France in 2006?	(Frischknecht and Stucki 2010)
BLCA	What is the maximum attainable affluence for the EU27 in 2020 and 2050 to meet related EU GHG target?	(Heijungs et al. 2014)
CLCA	What are the consequences of an increased demand of wheat in Denmark? Which effect does the decision to purchase an additional kWh of electricity have on the electricity market and/or on the environmental impacts?	(Schmidt 2010) (Frischknecht and Stucki 2010)
DLCA	Which effect does the decision to purchase an additional kWh of electricity have on the electricity market and/or on the environmental impacts?	(Frischknecht and Stucki 2010)
ILCA	What are the system-wide life-cycle impacts of a specific energy transition?	(Hertwich et al. 2014)
NLCA	What are the future environmental burdens associated with an emerging technology for both reasonable and extreme-case scenarios?	(Wender et al. 2014)
PLCA	What are the environmental benefits and impacts of nanosilver T-shirts compared with conventional T-shirts and T-shirts treated with triclosan?	(Walser et al. 2011)
SLCA	What is the best scenario for improving the life-cycle environmental performance of a car?	(Fukushima and Hirao 2002)

**Table 2:** Key characteristics of modes of LCA

	Question addressed	Key method	Object of analysis	Scope			Other methods/models used	Allocation method
				Temporal	Processes	Data		
ALCA	What are the environmental impacts of a product system as it currently functions?	LCA	Commercially existing product system; as it is or was	Present, past	All	SDb	n.a.	Variable
BLCA	What is a region’s maximum attainable affluence to meet its planetary boundaries at time *t* with constant technologies and population?	IOA	Regional/global consumption; as it should be	Future, past	All sectors		Linear programming (LP) simplex algorithm	Variable
CLCA	What are the consequences of an increased demand of a certain product system?	LCA	Commercially existing product system; as it changes due to a decision	Future	Marginal, market	SDb	CGEM; PGEM; IAM; LOM	Substitution
DLCA	What are the consequences of an increased demand of a certain product system?	LCA	Commercially existing product system; as it changes due to a decision	Future	Marginal, B2B	SDb	n.a.	Substitution
ILCA	What are the global life-cycle impacts of a specific energy transition?	LCA	Global energy consumption	Future	All	SDb and TI, B, IOA	IOA, IEA Blue Map scenario	Variable
NLCA	What are the expected environmental impacts of an emerging product system?	LCA	Emerging product system	Future	All	SDb and TI, F, B (optional)	Learning curves; technology and chemical models	Variable
PLCA	What are the expected environmental impacts of an emerging product system?	LCA	Emerging product system	Future	All	SDb and TI, F,B (optional)	Learning curves; technology and chemical models	Variable
SLCA	What are the expected environmental impacts of a certain future scenario of a product system?	LCA	Emerging product system	Dynamic, from past to future	All	Calculated	Life-cycle modeling language	Variable

*n.a.* not applicable; *product system* (or technology system) a set of unit processes interlinked by material, energy, product, waste, or service flows and performing one or more defined functions (Guinée et al. 2002); *SDb* standard LCA data(bases), such as ecoinvent, GaBi, ILCD, and USDA; *TI* assumptions on technical improvements in key energy and material production technologies (Hertwich et al. 2014); *F* foreground processes; *B* background processes; *CGEM* computable and partial general equilibrium model; *PGEM* partial general equilibrium model; *IAM* integrated assessment model; *LOM* linear optimization model; *All* all processes included for supplying the functional unit; *All sectors* all industry sectors included for supplying the region’s/global consumption; *Marginal* processes actually affected by the decision; *Market* affected processes are determined by using market information and price elasticities; *B2B* affected processes are determined by factual or anticipated economic business-to-business relationship

Table 2 shows that one main divide between modes concerns the object of analysis. Some modes mainly focus on commercially existing product systems (ALCA, CLCA, DLCA), while other modes focus on emerging, novel, and not yet marketed product systems (NLCA, PLCA, SLCA). Next, Table 2 shows that all modes except ALCA aim to assess the environmental life-cycle performance of a future system on the short, mid, or long term. CLCA then becomes just one mode out of at least six other modes to model life-cycle impacts of possible consequences of changes to existing product systems, or of introducing novel technology or product systems. Depending on the exact question posed, one of these six may be preferred, but we might also want to apply all of them and see how robust LCA results are for different assumptions that are difficult to verify or falsify (Yang and Heijungs 2018).

Another fundamental difference concerns the data requirements of the different modes: standard databases without any changes or standard databases with changes based on assumed technological improvements, supplemented by new datasets for foreground processes. As process data are one of the main drivers of final LCA results, differences at this level may have a huge effect on results.

Finally, Table 2 shows that by focusing the discussion on differences between modes, we may overlook the fact that there are also some fundamental modeling differences within one mode. This was recently exemplified for CLCA and argued to be potentially more important than differences between modes (Yang and Heijungs 2018). In addition, Table 2 shows comparable differences within NLCA and PLCA (see column “other methods/models used”).

## Conclusions

Over the past decade, an alphabet soup of modes of LCAs has emerged. This soup of LCA modes can be split into two main groups: ALCA is the only mode focusing on modeling a situation as it is, either in the past, present, or future, but without any changes. We suggest classifying the group of BLCA-ZLCA as life-cycle modeling of the unknown by exploring scenarios of potential futures, or shortly “explorative LCA.” Let us call them XLCA, where *X* ∈ {*B*, *C*, …, *Z*}, and moreover *X* codes for eXplorative.

Each mode of LCA has its merits and demerits and we have shown that they share many similarities despite also some differences. In addition, we have shown that the methods and models used in combination with the key method (i.e., mostly LCA) may widely vary. Therefore, based on an analysis of similarities and differences between the different XLCA modes, we conclude that rather than discussing different modes, the LCA community should focus on discussing the underlying differences including the object of analysis, other methods/models used in combination with the key method, data, and scenarios adopted. Our starting position in this debate is that instead of distinguishing BLCA-ZLCA as different modes of XLCA, we actually have a multi-model multi-paradigm approach within the group of XLCA (Yang and Heijungs 2018). Different questions posed will need different models and approaches and probably not just one model and one approach but rather a suite of applicable models and approaches. Selecting single models and approaches for specific questions may be a bridge too far. All models rely on strong assumption and constraints, and as LCA outcomes are largely unverifiable (Guineé et al. 2017; Yang and Heijungs 2018), claiming certain LCA modes or models to be superior cannot be supported by evidence, and this battle between schools is thus a dead-end in our view.

We strongly recommend that explorative LCA studies from here on explicitly formulate their research questions and object of analysis and justify other models, data, and scenarios selected on the basis of these questions and objects. We expect that the latter will help advance the debate on how different approaches, modes, and models may best support certain questions and decisions, which is a research topic on which little progress has been achieved so far.

We would like to conclude by paraphrasing Suh and Yang (2014): “Dividing the LCA world into CLCAs and ALCAs overlooks the studies” not fitting this divide and “hampers a constructive dialog about the creative use of modelling frameworks.”
